# Ultrasonographic Changes of Major Salivary Glands in Primary Sjögren’s Syndrome

**DOI:** 10.3390/jcm9030803

**Published:** 2020-03-16

**Authors:** Kyung-Ann Lee, Sang-Heon Lee, Hae-Rim Kim

**Affiliations:** 1 Division of Rheumatology, Department of Internal Medicine, Soonchunhyang University Seoul hospital, Seoul 04401, Korea; cyberag@naver.com; 2 Division of Rheumatology, Department of Internal Medicine, Konkuk University Medical Center, Research institute of medical science, Konkuk University School of Medicine, Seoul 05030, Korea; shlee@kuh.ac.kr

**Keywords:** Sjögren’s syndrome, salivary glands, ultrasonography, doppler, longitudinal studies

## Abstract

We aimed to evaluate the changes over time in salivary gland (SG) abnormalities by ultrasound (US) in patients with primary Sjögren’s syndrome (pSS). Patients with pSS (*n* = 70) and idiopathic sicca syndrome (*n* = 18) underwent baseline salivary gland ultrasound (SGUS) scans, and follow-up scans two years later. The semi-quantitative SGUS score (0–48) and intraglandular power Doppler signal (PDS) were assessed. We found that in the pSS group, the SGUS scores for total SGs and bilateral parotid glands significantly increased after the median 23.4-months follow-up. SGUS scores either worsened, improved, or were stable in 18.6%, 2.9%, and 78.6% of patients with pSS, respectively. The median changes from baseline in SGUS scores for total and parotid glands were +1.0 and +0.5, respectively. None of the SGUS scores changed significantly in the controls. The variables of homogeneity and hypoechoic showed a statistically significant progression of SGUS scores. In pSS patients, the baseline and follow-up PDS scores were significantly higher in the “worsening” group than in the “no change/improvement” group. Overall, the structural abnormalities in major SGs assessed using SGUS remained stable in patients with pSS. At the 2-year follow-up, SGUS scores worsened in 18.6% of patients with pSS. Intra-glandular hypervascularity was associated with the worsening of SG abnormalities.

## 1. Introduction

Primary Sjögren’s syndrome (pSS) is a systemic autoimmune disease characterized by lymphocytic infiltration of the exocrine glands resulting in dry eye and mouth [1]. Although long known to run a chronic and slowly progressive course in most patients, direct data on the natural history of glandular changes in pSS is insufficient. A few studies with sequential histopathologic evaluations of minor salivary glands (MSG) have been performed in patients with pSS. A 1992 study showed a significant increase in focus score after a mean interval of 3.5 years [2]. In addition, Jonsson et al. demonstrated that focus score increased significantly after mean 39 ± 20 months [3]. In contrast, during a median 55-month time interval of repetitive MSG biopsies (MSGB), the histological grades of inflammation in MSG remained almost unchanged [4].

A positive MSGB is a mandatory criterion for the classification of pSS in patients with absence of anti-SS-A/Ro [5]. Whether MSGB could detect a change sensitively, however, is uncertain. And the reproducibility and reliability of MSGB are also issues for its use as a biomarker [6]. Preferring its non-invasiveness and direct visualization of major salivary glands, a large number of studies have reported salivary gland ultrasound (SGUS) as a promising diagnostic tool [7,8]. Recently, two trials have used SGUS to monitor the therapeutic response to rituximab. These two trials demonstrated the improvement of SGUS scores after treatment with rituximab, suggesting SGUS as an imaging biomarker for pSS [9,10]. Despite this promise, evidence on the natural history of SGUS findings in patients with pSS is scarce. Although one study assessed the changes of SGUS over time, the number of patients was limited, and the follow-up SGUS was not performed routinely, leading to an irregular interval between the first and the second SGUS [11].

In this study, we aimed to evaluate the natural course of SGUS after a 2-year interval in patients with pSS and idiopathic sicca syndrome using the SGUS greyscale scoring system and intra-glandular power Doppler US (PDUS). The secondary aim was to assess the predictive factors of progressive changes in major salivary glands based on the SGUS scoring system.

## 2. Experimental Section

### 2.1. Study Population

This was a single-center prospective study performed at Konkuk University Medical Center, Korea, from March 2016 to February 2019. We enrolled patients with pSS and idiopathic sicca syndrome. The definitive diagnosis of pSS was made in accordance with the American–European Consensus (AECG) criteria [12]. Patients who did not fulfil the AECG criteria for pSS and received a diagnosis of idiopathic sicca syndrome, defined as non-immune mediated manifestations of dry eyes and mouth, served as controls. We excluded the patients with secondary Sjogren’s syndrome or pSS who received rituximab due to interference in investigating the time-course of SGUS changes. This study was conducted in accordance with the Declaration of Helsinki and approved by the Institutional Review Board for Human Research, Konkuk University Medical Center (KUH 1010776). The written informed consent was obtained from all participants.

### 2.2. Salivary Gland Ultrasonography Examination

The same examiner (L.K.A.) who was unaware of the clinical data performed SGUS on all patients. An HD15 US (Philips Ultrasound, Bothell, WA, USA) device with a linear 5–12 MHz probe was used. The bilateral parotid and submandibular glands were examined in the longitudinal and transverse planes. The patient was supine with the head slightly tilted opposite to the side being scanned to assess the parotid glands. Then the US of the submandibular glands was performed with the head maximally tilted back in a supine position. The thyroid was also scanned for evaluation of parenchymal echogenicity to compare to the salivary glands [13].

The semi-quantitative SGUS score (0–48) developed by Hocevar et al. [14] was used to investigate several aspects: (1) Parenchymal echogenicity compared to thyroid parenchyma, graded 0–1; (2) homogeneity, graded 0–3; (3) presence of hypoechoic areas in parenchyma, graded 0–3; (4) presence of hyperechoic foci, graded 0–3 in parotid glands and 0–1 in submandibular glands; (5) clearness of the salivary gland border, graded 0–3. The total ultrasound score was the sum of these five domains, ranged from 0 to 48. Since the cut-off value of 14 reproduced the best diagnostic accuracy in our previous work, [13] a score greater than 14 was defined as abnormal SGUS.

Intra-glandular PDUS was interpreted through a 4-grade semi-quantitative scoring system as follows: Grade 0 = no parenchymal flow, grade 1 = up to three single spots signals, or up to two confluent spots, or one confluent spot plus up to two single spots, grade 2 = flow signals in less than half of the cross-section of a gland (≤50%), and grade 3 = flow signals in more than half of the cross-section of a gland (>50%). The normal large vessels visible within the salivary glands were excluded from the PDUS score.

Enrolled patients underwent SGUS twice at baseline and 2 years (±2 months) later. The changes in these SGUS variables were evaluated in patients with pSS and idiopathic sicca syndrome.

### 2.3. Clinical and Laboratory Evaluation

Standardized clinical and laboratory evaluations were performed. For each patient, the following data were obtained and recorded: Patient demographics, history of dry eyes and mouth, and recurrent parotid enlargement, duration of subjective sicca symptoms, medication use, Schirmer’s test result, and whole unstimulated salivary flow rate. Enrolled patients underwent unstimulated salivary flow rate (USFR) tests twice at baseline and 2 years (±2 months) later, according to standardized methods [15]. To assess the disease activity at baseline, we measured the European League Against Rheumatism Sjögren’s syndrome disease activity index (ESSDAI) [16]. All patients completed the European League Against Rheumatism Sjögren’s Syndrome Patient-Reported Index (ESSPRI) to evaluate patient-reported dryness, fatigue, and pain [17].

Routine laboratory analyses were conducted at the first SGUS evaluation. Laboratory tests included white blood cell (WBC) count, lymphocyte, hemoglobin, and platelet concentrations, immunoglobulin G (IgG), and complement levels (C3, C4, CH50). Immunological testing included antinuclear antibody (ANA) (assessed on HEp-2 cells, a titer ≥1.160 was considered positive), anti-SS-A/Ro, and anti-SS-B/La (by enzyme-linked immunosorbent assay (ELISA)), and rheumatoid factor ((RF) by nephelometry) levels. Anti-SS-A/Ro and anti-SS-B/La testings were performed again at the time of follow-up SGUS.

### 2.4. Statistical Analysis

All statistical analyses were performed using the SPSS software package for Windows v. 17.0 (SPSS Inc., Chicago, IL, USA). A Mann-Whitney *U* and a Chi-squared test analyzed quantitative variables and categorical data, respectively. The SGUS scores between the two time points were compared using the paired Wilcoxon test. The improvement/worsening of SGUS scores was defined as a ≥1-point decrease/increase in the score. To identify SGUS items independently associated with worsening SGUS scores, we performed multivariate linear regression analyses. All SGUS domains that were associated with increased SGUS scores by univariate analysis with *p*-values less than 0.1 were entered into the multivariate model, applying backwards elimination. Results were considered statistically significant when *p* < 0.05.

## 3. Results

### 3.1. Baseline Characteristics of the Study Population

In total, 70 patients with pSS and 18 patients with idiopathic sicca syndrome completed the baseline and 2-year follow-up SGUS. Table 1 summarizes the baseline characteristics of the study population. No apparent differences were observed between the two groups regarding age, gender, duration of sicca symptoms, and proportion of patients with subjective and objective sicca symptoms.

As expected, all SGUS scores were higher in patients with pSS compared to patients with idiopathic sicca syndrome (*p* < 0.001). There were no significant differences between the two groups concerning the PDS sum scores of four salivary glands.

### 3.2. Changes in SGUS Scores over Time

**Figure 1:**
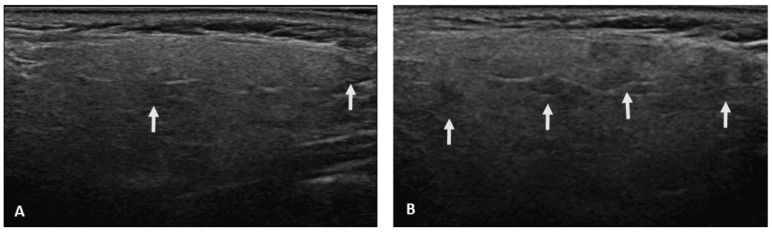
Ultrasonographic changes of the parotid gland after a 2-year follow-up. (**A**) The presence of hypoechoic areas (arrows) in the parenchyma increased from grade 1 (defined as few scattered) (**A**) to grade 2 (defined as several) (**B**).

Patients with pSS had shorter median interval time between the first and the second SGUS compared to patients with idiopathic sicca syndrome (median (IQR); 23.4 (2.7) vs. 25.4 (4.2) months) (*p* < 0.002). In the pSS group, the total SGUS scores and the SGUS scores for bilateral parotid glands significantly increased during the median 23.4-month follow-up (*p* = 0.013 and *p* = 0.011, respectively) (Table 2) (Figure 1). At follow-up, the absolute changes from baseline in total SGUS scores and SGUS scores for bilateral parotid glands in the pSS group were +1.0 and +0.5, respectively. The domains of homogeneity and hypoechoic areas showed a statistically significant progression of SGUS scores (β = 1.036, *p* < 0.001 and β = 0.736, *p* = 0.004, respectively). None of the SGUS scores changed significantly in patients with idiopathic sicca syndrome. The PDS sum scores of four salivary glands and USFR did not significantly change.

### 3.3. Comparison of Clinical, Laboratory, and SGUS Features in Relation to SGUS Scores 

We separated the patients into two groups based on whether SGUS scores worsened (*n* = 13), improved (*n* = 2), or showed no change (*n* = 55). Baseline and follow-up PDS sum scores of four salivary glands were significantly higher in the worsening SGUS group than no change/improvement SGUS group. No significant differences were observed between the two groups regarding age, duration of sicca symptoms, medication, serologic markers, USFR, ESSPRI, and ESSDAI (Table 3).

In the pSS group, 15 patients had an SGUS score <14 at baseline. Among them, SGUS scores changed in 2 patients (13.3%) after the follow-up study. One out of the 15 initially negative SGUS (SGUS score 13) was positive on the second US study (SGUS score 16). Another patient with increased SGUS scores (from 7 to 10) had negative SGUS in the initial and secondary US exams.

## 4. Discussion

The present longitudinal ultrasound study demonstrated the progressive course of major salivary glands in patients with pSS. Moreover, SGUS scores did not change significantly over 2 years in patients with idiopathic sicca syndrome. In contrast to our study, a recent study showed that SGUS abnormalities did not change significantly in both pSS and non-pSS patients during a mean of 1.9 years. However, the second SGUS was not a routine evaluation but was instead part of standard care by the managing physician, leading to follow-up intervals ranging from 1 year to 7 years. To avoid this variation, we investigated the SGUS scores at baseline then 2 years (± 2 months) later to confirm the natural course of major salivary glands. In the pSS group, salivary glandular involvement remained stable in a majority of patients, but 18.6% of patients had worsening SGUS scores, and the median absolute total SGUS scores changed 1.0 during the 2 years. Our findings suggested a slowly progressive course of salivary glandular involvement in patients with pSS.

We defined improvement or worsening of SGUS scores as a ≥1-point change in the score. Two randomized controlled trials also used SGUS for monitoring of the therapeutic response of rituximab in patients with pSS. Similar to our study, both trials also defined improvement in echostructure as a ≥1-point decrease in the score after 24 and 48 weeks of treatment [9,10]. However, one-point changes in SGUS scores could be marginal and insufficient to provide clinical significance. Therefore, longer longitudinal studies are needed to reveal sensitivity to changes in SGUS scores in patients with pSS.

To date, a diversity of SGUS scoring systems have been developed, aiming to improve the diagnosis of pSS. The most relevant SGUS feature is parenchymal inhomogeneity [18]. However, there are other detectable SGUS abnormalities, such as decreased echogenicity, hyperechoic bands, calcifications, size variations of hypo-anechoic areas, intraglandular lymph nodes, irregularities of glandular borders, and hypervascularity [19]. Since we assumed that only evaluating the homogeneity and hypoechoic areas could be insufficient to reflect the time course of SGUS in pSS, the present study used the semi-quantitative SGUS score (0–48). Five domains comprise the 0–48 SGUS scoring system, including not only homogeneity and hypoechogenic areas, but also parenchymal echogenicity, clearness of posterior border margin, and hyperechogenic reflections [14]. We examined which domains were associated with the progression of SGUS scores. We found significant changes in inhomogeneity and hypoechoic areas over 2 years, which are US hallmarks of pSS. Changes in the other SGUS domains, however, were not observed. The two-year follow-up period could be a relatively short period to describe the whole longitudinal features of SGUS in pSS. Moreover, the corresponding histopathologic features of each SGUS variable are unknown. Which US finding represents irreversible (damage), or reversible (inflammation) findings remain questionable [18]. Therefore, studies integrating US and histopathology of salivary glands are needed to determine the sensitive markers of SGUS over time.

The power Doppler (PD) signals represent the status of increased blood flow and active inflammation. Although increased PD signals of synovitis were reported to be associated with radiographic progression in rheumatoid arthritis [20], evidence suggesting a connection between increased PD signals and prognosis of salivary glands is lacking in pSS. We found that pSS patients with worsening SGUS scores had higher baseline and follow-up PDUS scores compared to patients with no change or improvement of SGUS scores. In our previous work, we described reduced PDUS scores in patients with advanced pSS compared to pSS patients without definite SGUS structural abnormality, suggesting that hypervascularity reflecting the inflammatory phase of pSS could lead to glandular hypovascularity in late stages of pSS [13]. Together, our present and previous studies indicate that intraglandular hypervascularity could result in the structural progression of salivary glands in patients with pSS. However, without explicit data directly correlating intra-glandular PD signals with active inflammation, further studies comparing histopathologic findings (vascularity and inflammation) and PDUS are needed. In addition, this study showed that the PDS scores in the worsening group did not change at the follow-up when the median value of SGUS scores became comparable to those of the no change/improved group. Considering the slowly progressive course of pSS, glandular hypervascularization in the inflammatory phase could be unaltered and last more than 2 years. A long-term longitudinal study evaluating changes in PDS scores of salivary glands is needed to reveal the duration of glandular vascularity and confirm the link between hypervascularity and progression of parenchymal abnormalities of salivary glands.

To assess the factors associated with glandular progression in patients with pSS, various clinical, laboratory, and SGUS features were compared between worsening and no change/improved SGUS groups. However, prognostic factors for pSS, such positive autoantibody titers, levels of complement, and ESSDAI were not different between the two groups. Only PDS sum scores showed a significant difference between the two groups. Although well-designed randomized, controlled trials (RCT) providing evidence for the role of Hydroxychloroquine (HCQ) in pSS are insufficient, current published studies showed that the efficacy of HCQ in pSS was not superior to placebo in treating dry mouth or dry eyes in pSS patients [21]. Corroborating this, one RCT showed no significant difference in USFR between patients treated with HCQ and placebo [22]. Inconsistent with the previous studies, we also confirmed that the progression of major salivary glands in pSS measured by SGUS scores was not associated with the use of HCQ.

The prevalence of anti-Ro/SSA is 50–70% in pSS, and anti-Ro/SSA and anti-La/SSB correlate with younger age at diagnosis, more severe dysfunction of the exocrine gland, and higher intensity of the exocrine glands [23]. In our previous work, double seropositivity of anti-Ro/SSA and La/SSB was independently associated with high SGUS scores in pSS [13]. Utilizing this previously-published data, we further examined the changes in anti-Ro/SSA and La/SSB after 2 years to assess the association with progressive glandular involvement of pSS. No association between seroconversion and changes of SGUS scores was found. Negative seroconversion of anti-Ro/SSA and anti-La/SSB were observed in 14.2% and 8.6% of patients with pSS, respectively. Positive seroconversion of anti-Ro/SSA and anti-La/SSB was rarely observed (0% and 1.4%, respectively). The frequent fluctuation of anti-extractable antigens (anti-ENA) in systemic lupus erythematosus was reported in a longitudinal study, showing 30–50% of negative and 14.7–56% of positive seroconversion of anti-ENA [24]. Compared to the SLE study, we found less anti-Ro/SSA and anti-La/SSB fluctuations over time in patients with pSS.

We observed that USFR did not change significantly in both pSS and idiopathic sicca syndrome groups. A previous study reported that stimulated salivary flow rates (SSFR) significantly dropped at a mean of 3.6 years of follow up in patients with pSS, although no alteration of USFR was observed [25]. On the other hand, Jonsson et al. described a mean follow-up of 39 months with 21 pSS patients and showed no reduction in stimulated whole salivary secretion [3]. Salivary gland dysfunction is a key manifestation of pSS and evaluation of USFR is a diagnostic criterion for pSS [5]. However, USFR showed low sensitivity (52%) in establishing the diagnosis of pSS [26], and the USFR test is relatively dependent on drug effects and environmental factors compared to SSFR [3]. The weak test-retest reliability of USFR has been reported [27]. Therefore, the USFR may not be suitable for monitoring glandular function. Further studies using both USFR and SSFR are needed to investigate the long-term natural course of salivary gland function in patients with pSS.

While providing insight into pSS progression, the present study has some limitations. The number of patients with idiopathic sicca syndrome was relatively small, and the data on changes in SGUS scores in healthy controls without dry mouth and SSFR were absent. Furthermore, this study was a single-center study and a single expert performed SGUS at a 2-year follow up. Although the homogeneity item showed good inter-observer reliability [28], we could not calculate the inter-observer agreement for each item in this study. Since SGUS scores barely worsened over a short follow up, long-term multicenter studies with a reliability exercise are needed to verify our study results and reveal the meaning of changes in SGUS scores.

## 5. Conclusions

In conclusion, the structural abnormalities in major salivary glands assessed using SGUS scores remained stable in the majority of patients with pSS. During the 2-year study, SGUS scores progressed in 18.6% of patients with pSS. The homogeneity and hypoechoic areas are domains that showed significant progression. Intra-glandular hypervascularity was associated with the worsening of salivary gland abnormalities, providing a potential predictive marker for glandular progression in pSS.

## Figures and Tables

**Table 1:** Baseline characteristics of the study population.

	pSS (*n* = 70)	Idiopathic Sicca Syndrome (*n* = 18)	*p*-Value
Age, median (IQR), years	57.6 (18.0)	65.5 (6.0)	0.053
Female, *n* (%)	67 (95.7)	17 (94.4)	1.000
Duration of sicca symptoms, median (IQR), years	5.0 (7.0)	5.5 (7.0)	0.299
Xerostomia, *n* (%)	68 (67.1)	18 (100.0)	1.000
Xerophthalmia, *n* (%)	65 (92.9)	14 (77.8)	0.080
Abnormal Schirmer’s test, *n* (%)	69 (98.6)	18 (100.0)	1.000
USFR, median (IQR), mL/15 min	1.3 (2.0)	2.75 (2.28)	0.247
Abnormal USFR, *n* (%)	41 (58.6)	6 (33.3)	0.056
Positive ANA, *n* (%)	50 (71.4)	1 (5.56)	<0.001
Positive anti-Ro/SSA, *n* (%)	64 (91.4)	0 (0)	<0.001
Positive anti-La/SSB, *n* (%)	35 (50.0)	0 (0)	<0.001
Positive RF, *n* (%)	31 (44.3)	7 (38.9)	0.743
RF, median (IQR), IU/dL	27.0 (31)	15.0 (32)	0.392
IgG, median (IQR), mg/dL	1801.0 (440)	1546.1 (485)	0.010
C3, median (IQR), mg/dL	94.5 (9.3)	98.8 (23.8)	0.011
C4, median (IQR), mg/dL	25.1 (10.0)	23.0 (9.0)	0.108
Total SGUS scores at baseline, median (IQR)	27 (14)	4 (3)	<0.001
Total SGUS scores≥14 at baseline, *n* (%)	55 (78.6)	1 (5.6)	<0.001
Total SGUS scores for the parotid glands at baseline, median (IQR)	12.0 (10)	2.0 (2)	<0.001
Total SGUS scores for the submandibular glands at baseline, median (IQR)	14.0 (9)	2.0 (4)	<0.001
PDS sum scores of four salivary glands, median (IQR)	0 (3)	1 (3)	0.320

pSS: Primary Sjögren’s syndrome; IQR: Interquartile range; USFR: Unstimulated salivary flow rate; ANA: Antinuclear antibody; SSA: Sjögren’s-syndrome-related antigen A, SSB: Sjögren’s-syndrome-related antigen B; RF: Rheumatoid factor; Ig: Immunoglobulin; C: Complement; SGUS: Salivary gland ultrasound; PDS: Power Doppler signal.

**Table 2:** Changes in SGUS scores over time.

	pSS (*n* = 70)			Idiopathic Sicca Syndrome (*n* = 18)			pSS vs. Idiopathic Sicca Syndrome at Follow-Up
	Baseline	Follow-up	*p*-Value	Baseline	Follow-up	*p*-value	*p*-value
Total SGUS scores	27 (14)	28 (15)	0.013	4 (3)	4.5 (4)	0.157	<0.001
Total SGUS scores ≥14 at baseline, *n* (%)	55 (78.6)	56 (80.0)	1.000	1 (5.6)	1 (5.6)	1.000	<0.001
SGUS scores for the parotid glands	12 (10)	12 (9)	0.011	2 (2)	2 (2)	1.000	<0.001
SGUS scores for the submandibular glands	14 (9)	14 (8)	0.154	2 (4)	2 (4)	0.157	<0.001
PDS sum scores of four salivary glands	0 (3)	0 (3)	1.000	2 (3)	2 (2)	0.786	0.303
USFR, mL/15 min	1.3 (2.0)	1.0 (1.67)	0.086	2.75 (2.28)	2.5 (6.9)	0.285	0.073

Data are expressed as median (IQR). *p*-values in bold are significant. pSS: Primary Sjögren’s syndrome; IQR: Interquartile range; SGUS: Salivary gland ultrasound; PDS: Power Doppler signal, USFR: Unstimulated salivary flow rate.

**Table 3:** Clinical, laboratory, and SGUS features according to changes in SGUS scores.

	Worsening (*n* = 13)	No Change or Improved (*n* = 55/2)	*p*-Value
Age, years	62.0 (16)	57.0 (19)	0.197
Female, *n* (%)	13 (100)	54 (94.7)	1.000
Duration of sicca symptoms, years	5.0 (8)	5.0 (6.0)	0.058
Interval from 1st to 2nd SGUS, months	24.2 (2.4)	23.0 (2.8)	0.369
Xerostomia, *n* (%)	13 (100)	55 (96.5)	1.000
Xerophthalmia, *n* (%)	12 (92.3)	53 (93.0)	1.000
Abnormal Schirmer’s test, *n* (%)	13 (100)	56 (98.2)	1.000
Baseline USFR, mL/15 min	2.2 (2.7)	1.3 (2.0)	0.932
Follow-up USFR, mL/15 min	3.0 (3.25)	1.0 (1.5)	0.606
Current medication			
Hydroxychloroquine (HCQ), *n* (%)	11 (84.6)	44 (77.2)	0.720
HCQ dose, mg/day	200 (150)	200 (200)	0.877
Corticosteroids *n* (%)	3 (23.1)	12 (21.1)	1.000
Dose of corticosteroids, mg/day	0 (1.25)	0 (0)	0.950
Azathioprine, *n* (%)	1 (7.7)	6 (10.5)	1.000
Positive ANA, *n* (%)	12 (92.3)	38 (66.67)	0.091
Positive anti-Ro/SSA, *n* (%)	11 (84.6)	53 (93.0)	0.308
Positive anti-La/SSB, *n* (%)	8 (61.5)	27 (47.4)	0.540
Negative seroconversion anti-Ro/SSA at follow-up, *n* (%)	1 (7.7)	9 (15.8)	0.675
Negative seroconversion of anti-La/SSA at follow-up, *n* (%)	1 (7.7)	5 (8.8)	1.000
Positive seroconversion of anti-Ro/SSA at follow-up, *n* (%)	0 (0)	0 (0)	-
Positive seroconversion of anti-La/SSA at follow-up, *n* (%)	0 (0)	1 (1.8)	1.000
RF, IU/dL	15 (31)	27 (30)	0.907
IgG, mg/dL	1948 (607)	1546 (499)	0.107
C3, mg/dL	92.8 (7.0)	97.6 (26.1)	0.123
C4, mg/dL	20.3 (9.3)	23.0 (8.7)	0.874
ESSDAI (123 = maximal disease activity)	4.0 (4.0)	2.0 (3.0)	0.225
ESSPRI (10 = maximal symptom severity)	6 (4.35)	5.7 (3.3)	0.606
Total SGUS scores at baseline	24 (12)	29 (15)	0.634
Total SGUS scores ≥14 at baseline, *n* (%)	11 (84.6)	44 (77.2)	0.720
Total SGUS scores at follow-up	27 (11)	28 (15)	0.487
Total SGUS scores ≥14 at follow-up, *n* (%)	12 (92.3)	44 (77.2)	0.441
Total SGUS scores for the parotid glands at baseline	11 (7)	12 (11)	0.838
Total SGUS scores for the submandibular glands at baseline	14 (7)	14 (10)	0.617
Total SGUS scores for the parotid glands at follow-up	13 (6)	12 (11)	0.57
Total SGUS scores for the submandibular glands at follow-up	16 (4)	14 (10)	0.585
Baseline PDS sum scores of four salivary glands	2 (3)	0 (2)	0.008
Follow-up PDS sum scores of four salivary glands	2 (3)	0 (2)	0.008

Value are presented as median (IQR) unless otherwise stated. *p*-values in bold are statistically significant. SGUS: Salivary gland ultrasound; pSS: Primary Sjögren’s syndrome; IQR: Interquartile range; USFR: Unstimulated salivary flow rate; ANA: Antinuclear antibody; SSA: Sjögren’s-syndrome-related antigen A, SSB: Sjögren’s-syndrome-related antigen B; RF: Rheumatoid factor; Ig: Immunoglobulin; C: Complement; ESSDAI: European League Against Rheumatism Sjögren’s Syndrome Disease Activity Index; ESSPRI: European Sjögren’s Syndrome Patient Reported Index; PDS: Power Doppler signal.
